# Sustainability aspects of food and drinks offered in vending machines at Slovenian universities

**DOI:** 10.3389/fnut.2025.1439690

**Published:** 2025-06-23

**Authors:** Urška Rozman, Mateja Lorber, Anja Bolha, Jasmina Bevc Bahar, Miha Lavrič, Sonja Šostar Turk

**Affiliations:** ^1^Faculty of Health Sciences, University of Maribor, Maribor, Slovenia; ^2^Slovene Consumers’ Association, Ljubljana, Slovenia

**Keywords:** vending machines, university, food nutritional quality, sustainability, environmental impact

## Abstract

**Introduction:**

Vending machines offer a convenient way for food distribution, particularly favored by employees, students, and individuals seeking a quick snack. Food vending machines typically offer unhealthy, calorie-dense, and nutrient-poor options, which contribute to the rise of non-communicable diseases. Creating a healthier food environment is crucial, particularly in universities where students are developing their eating habits and becoming more independent. Key considerations for vending machines include the quality, nutritional value, and price of the products with a recent and growing attention toward sustainability.

**Methods:**

The present study thoroughly examined 30 vending machines across 30 faculties in Slovenia. The analysis focused on assessing the variety and sustainability of the available products. The following was evaluated through three primary criteria, based on the information available on the product label: nutritional quality, environmental impact (palm oil content, packaging materials, and sustainability certificates), and socioeconomic indicators (suitability for people with special dietary needs).

**Results:**

The results revealed a low proportion of products met the proposed sustainability criteria, highlighting the need to promote sustainability in the vending machine industry. Although food categories like dairy products, fruits, and nuts have better nutritional profiles, they are underrepresented. In contrast, items like biscuits, crisps, snacks, and pre-prepared sandwiches often exceed recommended fat, salt, and sugar levels. More than one-quarter of products contained palm oil, only two were labeled as palm oil-free, and a limited proportion of products were suitable for individuals with special dietary requirements such as gluten sensitivity and lactose intolerance.

**Discussion:**

Improving the food selection in vending machines, guided by suggested sustainability criteria, presents a promising strategy for reshaping the food environment and promoting sustainable healthy diets, taking into account nutritional, environmental, and socioeconomic indicators.

## 1 Introduction

According to WHO Guiding Principles (1), sustainable healthy diets should promote individual health, have a low environmental impact, and consider sociocultural aspects. To promote the health of individuals, the intake of nutritionally adequate, unprocessed foods is emphasized (1). Therefore, the nutritional quality of food is recognized as an essential dimension of a sustainable healthy diet (2). As an important factor influencing the intake of nutritionally adequate, unprocessed food, food marketing, and distribution should also be considered. In this regard, vending machines are an important factor in achieving sustainable goals. Usually, food vending machines offer unhealthy, energy-dense, and nutrient-poor foods (3–7), contributing to the global burden of non-communicable diseases (8–11). It is necessary to provide a healthy food environment, especially in universities with students as young adults who are becoming independent and developing their eating habits (12). Due to various factors, university students are at risk of developing poor eating habits, leading to malnutrition or over nutrition and increasing the risk of preventable diseases (13). Many students consume diets deficient in essential nutrients and consume foods that are high in calories, sugar and fat (13). Likewise, Slovenian students do not pay too much attention to healthy food choices, which shows that they are not yet aware of the importance of a healthy diet for strengthening and maintaining health (14). Students often do not have the opportunity, time and enough financial resources for a hot and healthy meal, especially at those faculties that do not offer a student canteen. In such cases, the vending machine is the only option for buying a snack or lunch (15, 16). Since food vending machines were recognized as an obesogenic food environment at universities (17), positively associated with higher consumption and greater frequency of snacks (18), there have been several attempts and nutrition interventions aimed at increasing the availability and consumption of healthy products (19–22). Fewer studies focused on the sustainable aspect of foods offered in vending machines (23, 24), but the European Vending Machine Association has recognized sustainability as an important aspect and is already preparing activities for sustainable vending (25). In 2015, Slovenia adopted the National Program on Nutrition and Physical Activity for Health 2015–2025 (Dober tek, Slovenija - eng. Enjoy your meal Slovenia). According to guidelines and recommendations, students are placed in one of the priority areas for ensuring healthy eating (26). As a part of the program, Recommendations for a healthy choice in vending machines (27), were developed by experts and confirmed by the Ministry of Health. Recommendations were first based only on the nutrition quality of the food products, but are currently being revised to give special emphasis also to other sustainability elements.

Sustainability labeling (presence of certificates) is one of the features that inform the consumer about the sustainability of a food product. They often take the form of a logo and/or a statement on the product that informs the consumer about the standard of the respective system that the product meets, e.g., that it has been produced organically, that it has been produced with consideration for the local economy (e.g., to avoid poverty) or in compliance with additional requirements relating to animal welfare (28). The presence of palm oil in processed food products is also identified as an indicator of an unhealthy and unsustainable food product by consumers (29). Palm trees are produced in monocultures, causing deforestation and other types of environmental degradation in tropical regions of Asia, Africa, and Latin America, therefore palm oil production is considered unsustainable (30).

## 2 Materials and methods

### 2.1 Data collection

A cross-sectional survey of vending machines offering foods in faculties across Slovenia was conducted in April–May 2023. A convenience sample of 30 faculties from three main Slovenian public Universities (Ljubljana, Maribor, and Primorska) was chosen, representing half of all Slovenian public faculties. Some faculties had several vending machines present, but we only collected product data on one vending machine at each faculty, namely the one that was positioned on the most frequently visited part of the faculty, based on observation of students. All vending machines were cooled, so all providers had the same product placement conditions. We did not include machines for preparing hot drinks in the research. The information about the faculty and vending machine provider was recorded for each vending machine, along with the number of full slots and total number of slots. The number of all (full and empty) slots was used to calculate the percentages of products in each product group based on total number of slots. Data collection was based on face-front items in each vending machine, i.e., an item in a slot next in line to be sold (31). All the different products present in the vending machines were purchased so that we could obtain the necessary information. According to the Global Food Monitoring Group food categorization system (32), all identified foods were assigned to one of the 14 food categories. Detailed information including product name, manufacturer, net quantity, ingredient list, price, and the number of occupied slots in the vending machine (spirals) was recorded for each unique product label. Nutritional value per 100 g, including energy value, fat, saturated fatty acids, carbohydrates, sugars, salt, protein, and dietary fiber, was recorded for each unique product label. The data on the nutritional composition of the food products were obtained solely from the labels of the collected products. The ingredient list was screened to identify any presence of palm oil. Additionally, observations were made regarding the type of packaging and the presence of sustainability labeling (certificates), for each product. The socioeconomic aspect of sustainability was observed through the availability of products for people with special dietary needs, namely gluten sensitivity and lactose intolerance. Acceptability, including physical adaptations such as digestibility and intolerance, is recognized as one of the socioeconomic indicators for a sustainable healthy diet (2) and the demand for gluten- and lactose-free products has greatly increased in recent years (33).

### 2.2 Data processing and analyses

Data was collected and processed using Microsoft Excel 16.0. (Microsoft Corporation, Redmond, United States). The variety of the vending machines products was assessed as the presence of different food categories within the vending machines so that each product was assigned to the appropriate food category according to Table 1. The median, Q1, Q3, lower, and upper bounds, as well as outliers were calculated and displayed as a box and whisker plot. The selection of sustainability elements, assessed and evaluated in this study, was based on WHO guiding principles for sustainable healthy diets. Elements of food products found in vending machines, such as nutritional value, packaging type, presence of sustainability certificates and presence of palm oil were most clearly identified with at least one of the 14 sustainability dimensions listed (1). To assess the proportion of sustainable products within each food category, the nutritional quality of products was initially assessed. A maximum acceptable amount of fat, sugar, salt, and minimum amount of dietary fiber was determined (Table 1) based on “Recommendations for a healthy choice in vending machines,” prepared within the national program “Dober Tek Slovenija” (eng. Enjoy your meal Slovenia), the project “Prava izbira – veš kaj ješ” (eng. “The right choice – know what you eat”) and confirmed by the Ministry of health of the Republic of Slovenia (27). Products meeting all of the nutritional quality criteria in Table 1, were designated as sustainable. The second sustainability criterion focused on the type of packaging and the food sustainability labeling. Packaging materials were classified as sustainable if they comprised recycled plastic or FSC cardboard. In addition, return packaging was also classified as a sustainable attribute. Certificates considered as indicators of sustainability were EU green leaf (ecological product), Fairtrade, and Rainforest Alliance. The presence of palm oil as an ingredient was considered the third aspect of sustainability, with products containing palm oil being classified as less sustainable. Finally, the shares of gluten- or lactose-free labeled items as part of the total, beverage, and non-beverage offerings, were compared.

**TABLE 1:** Nutritional criteria for food categories.

(Sub)category	Fat (100g/100 mL)	Sugar (100g/100 mL)	Salt (100g/100 mL)	Dietary fiber (100 g)
Biscuits (biscuits, cookies, croissant, wafer)	20	20 for biscuits and cookies, 15 for croissants and wafer	1.5	/
Cereal bars	20	15	1.5	> 3
Chocolate and sweets (candy, chocolate, chocolate/chocolate snacks)	/	15	1.5	> 3
Crisps and snacks (chips, crackers, flips, salted sticks/salty pretzels)	20	15	1.5	> 3
Fruit (fresh fruits, dried fruits, fruit sticks)	/	**	1.5	/
Milk	/	**	0.3	/
Nuts and seeds	/	/	1.5	/
Pre-prepared Salads and Sandwiches**	20	/	1.5	> 3
Yogurt products	/	10	0.3	/
Soft drinks*	/	**	/	/
Waters*	/	**	/	/
Coffee*	/	**	/	/
Juices*	/	**	/	/
Energy drinks*	/	**	/	/

*No added sweeteners. **No added sugar.

## 3 Results

**FIGURE 1:**
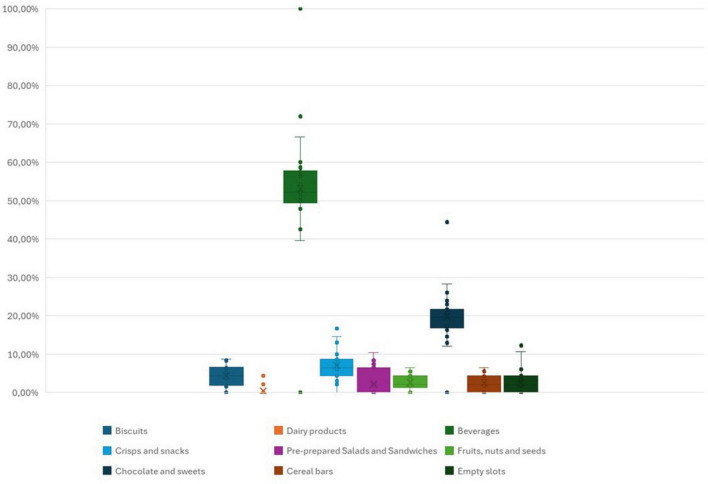
Variety of food categories within the vending machines.

The 30 different surveyed vending machines were filled and serviced by four providers, each contributing to varying extents. Provider 1 supplied 21 machines (70.00% of total surveyed machines), provider 2 supplied seven machines (23.33%), while providers 3 and 4 were each present at single faculties (3.33% per provider). In the examined vending machines, drinks (54.00%) i.e., soft drinks, water, juices, and energy drinks, were the most represented food category. This was followed by the food category of chocolates and sweets, representing 22.26% of the assortment. Food category biscuits constituted approximately 10.32% of the product inventory, while crisps and snacks comprised a slightly smaller proportion at 8.52%. Further analysis revealed the presence of next food categories: cereal bars (3.05%), pre-prepared salads and sandwiches (2.87%), as well as nuts and seeds (2.34%), in comparable quantities. The least common food category were dairy products such as milk and yogurt (0.53%), and fruits (0.53%). The variety of food categories within the surveyed vending machines is shown in Figure 1.

To assess the differences between individual vending machines, we analyze the representation of nutritionally more favorable food categories (i.e., cereal bars, salads, nuts, seeds, and dairy products). Out of 30 vending machines, seven vending machines offer between 9% and 15% of nutritionally more favorable food categories, with the highest percentage of representation being in the Faculty of Medicine. In eight vending machines the offer of nutritionally more favorable food categories was 6%–7% and 13 vending machines offer 2%–4% of nutritionally more favorable food categories. There were also 2 vending machines where none of the nutritionally more favorable food categories were present.

### 3.1 Nutritional quality

**FIGURE 2:**
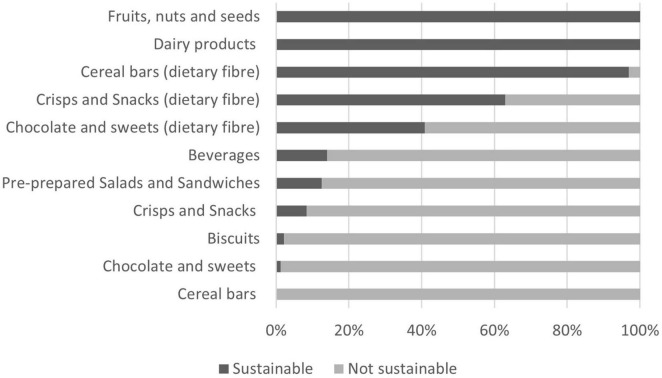
Share of sustainable products by food category.

In Figure 2 the shares of sustainable products by food category are presented, as determined by the nutritional criteria outlined in Table 1. Notably, the predominant category occupying the majority of vending machine slots was beverages, comprising 54.34% of the total offerings. Within the beverage category, sweetened drinks, encompassing colas, iced teas, and similar variants, constituted the largest share, accounting for 49.34% of all beverages. A further breakdown revealed that 17. 46% of the beverages consisted of water variants (plain, mineral, carbonated), while 14.68% comprised low-calorie non-alcoholic beverages containing added sugars or sweeteners. Additionally, 7.28% of the beverage assortment consisted of energy drinks, 3.31% of juices and smoothies, 3.17% of nectars and fruit drinks, 3.31% of sweet drinks without energy value, and 1.46% of low-calorie non-alcoholic drinks with no added sugar and/or sweeteners. According to the nutritional quality criteria in Table 1, only 13.99% (82 of 586) of the available products among beverages met the criteria for sustainability, meaning there was no added sugar or sweeteners.

From the “biscuits” category, which represented 9.82% of the entire offer, the most common were croissants (54 slots) and cookies (52 slots). Additionally, cakes were present in 19 slots and wafers in 12 slots. Interestingly, no bread products were identified across any of the examined vending machines. Only three products within the biscuit category met the criteria for sustainability based on their nutritional quality, these were: vanilla cake, walnut cake, and cottage cheesecake. The rest of the products were excessively fatty (containing more than 20 g of total fat per 100 g) or excessively sweet (containing more than 15 g of total sugar per 100 g).

Cereal bars, identified in 22 of the 30 vending machines surveyed, accounted for 2.37% of the overall product inventory. All cereal bars had a sugar content higher than specified in Table 1, containing more than 15 g of total sugars per 100 g. However, all cereal bars had more than 3.0 g of dietary fiber per 100 g of product, presenting a potential exception for consideration as a sustainable option based on their nutritional profile. However, none of the examined cereal bars were suitable for people with gluten sensitivity.

Chocolate and sweets represented 18.63% of the offer in vending machines, including a variety of items such as filled chocolate, chocolate snacks, chocolate protein bars, etc., Notably, only three individual products within this category contained less than 15 g of sugar per 100 g. However, when considering the dietary fiber content, 40.91% could exceptionally be considered sustainable.

Within the “Pre-prepared Salads and Sandwiches” category, only sandwiches were available in surveyed vending machines. They were found in nine of the 30 examined machines, representing 2.37% of the total product assortment. Assessment based on the nutritional criteria outlined in Table 1 revealed that 56.25% of these sandwiches met the standards concerning salt content. However, it is noteworthy that in the majority of sandwiches, specifically 28 out of 32, the presence of added sugar and/or sweeteners was identified.

Dairy products were notably sparse within the surveyed vending machines, present in only three of the 30 machines, indicating a limited supply in this category. Plain milk was available in just one section, accompanied by plain yogurt with 3.2% milk fat in four sections, and fruit yogurt in one section. All products from this category can be characterized as sustainable according to the values in Table 1.

Products from the “fruits” and “nuts and seeds” categories appeared in 22 out of 30 surveyed vending machines, collectively comprising 2.44% of the total product assortment. Among these, nuts, dried fruits, and nut mixtures were present in 27 slots. Apples were found only in two slots, freeze-dried pineapple in one slot, and fruit sticks in four slots. All examined products within these categories met the criteria defined in Table 1.

Crisps and snacks represented 7.83% of the total offer. Among snacks, chips, and flips were the most prevalent (50 slots), followed by breadsticks (23 slots), and crackers (22 slots). The least offered were corn waffles and salted nuts, each occupying one slot. Regarding nutritional criteria in Table 1, only 8.42% met the standards for crisps and snacks. However, 63.16% of products contained more than 3.0 g of dietary fiber per 100 g.

### 3.2 Packaging type, presence of certificates, and palm oil

**FIGURE 3:**
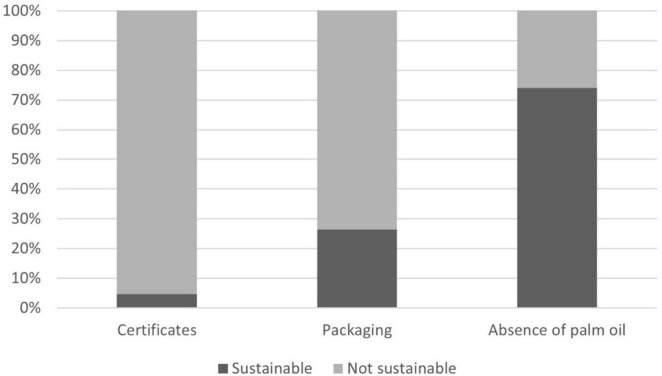
Share of sustainable products regarding packaging, presence of certificates, and absence of palm oil.

As the second criterion for assessing product sustainability, the environmental impact of products was defined based on the information available on the product label. First, the packaging type and the presence of sustainability certificates were evaluated (see Figure 3). Of all products, 26.38% were identified as being packed in sustainable materials, such as recycled plastic, FSC cardboard, or return packaging. The proportion of products with food production-related certificates (e.g., Fair-trade cacao, Rainforest Alliance, EU organic logo) was much lower, accounting for only 4.70% of the total offer. The presence/absence of palm oil was also considered, as illustrated in Figure 3. Among all products analyzed, 25.85% contained palm oil and were thus classified as less sustainable. On the contrary, only two products were labeled as palm oil-free.

**FIGURE 4:**
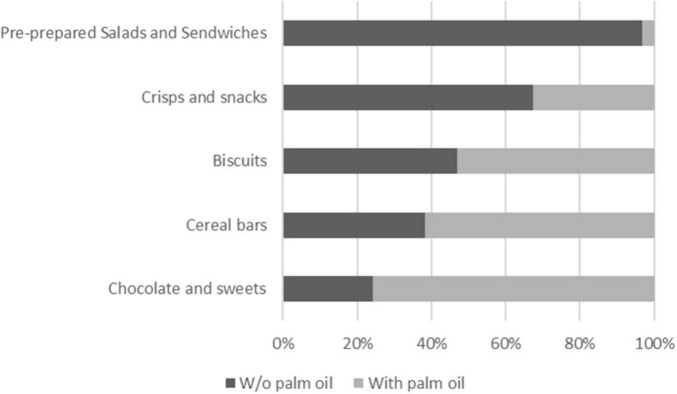
Share of products with/without palm oil in food categories: biscuits, cereal bars, chocolate and sweets, crisps and snacks, pre-prepared salads and sandwiches.

Since there are several food categories where palm oil is not expected, we analyze the presence of palm oil in the food categories biscuits, cereal bars, chocolate and sweets, crisps and snacks, pre-prepared salads and sandwiches (Figure 4).

As expected, in all mentioned categories, palm oil was present in 3.13% (pre-prepared salads and sandwiches) up to 75.80% (Chocolates and sweets) of the products.

### 3.3 Products for people with special dietary needs

**FIGURE 5:**
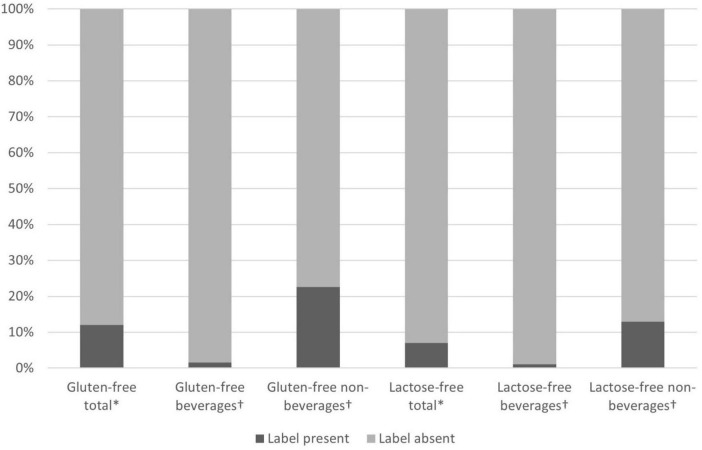
Share of gluten- and lactose-free labeled products of total, beverage, and non-beverage offers. *Percentage of total offer. ^†^ Percentage of beverage or non-beverage (including milk and dairy) offers.

When evaluating the nutritional aspect of offered products, the presence or absence of gluten-free or lactose-free labels was recorded (see Figure 5). In total, 12.04% of products were labeled gluten-free and 7.01% lactose-free. When looking only at non-beverage (incl. milk and dairy products) items, which comprised 49.55% of the total offer, 22.63% were labeled as gluten-free, and 12.98% as lactose-free. No milk and dairy products were labeled either as lactose- or gluten-free. In the case of offered beverages, comprising 50.45% of the total offer, 1.63% were labeled gluten-free, and 1.14% were lactose-free.

**FIGURE 6:**
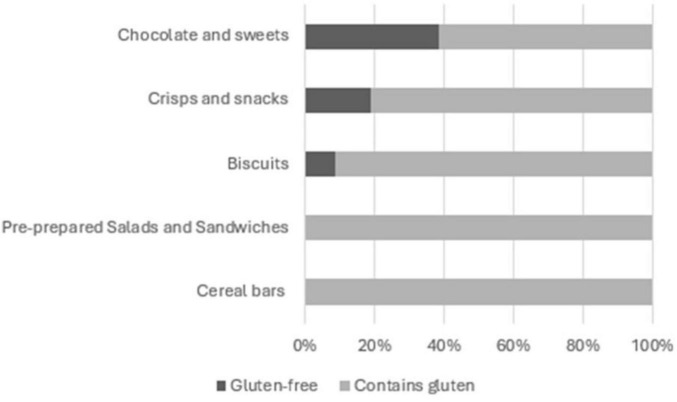
Share of gluten-free labeled products within food categories pre-prepared salads and sandwiches, cereal bars, chocolate and sweets, crisps and snacks and biscuits.

**FIGURE 7:**
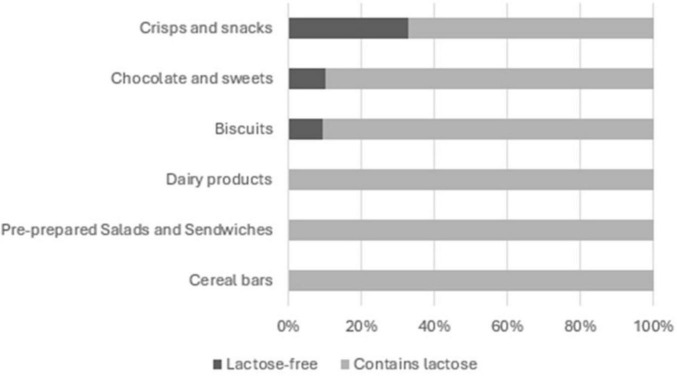
Share of lactose-free labelled products within food categories pre-prepared salads and sandwiches, cereal bars, chocolate and sweets, crisps and snacks, biscuits and dairy products.

As there are several food categories where gluten and/or lactose are not expected, we analyze the presence of those in only selected food categories (Figures 6, 7). Regarding gluten, it was present in all products from food categories Pre-prepared Salads and Sandwiches and Cereal bars, but also in 61.69% of Chocolate and sweets, in 81.05% of Crisps and snacks and in 91.30% of Biscuits (Figure 6).

Lactose was present in all the products from food categories: Cereal bars, Pre-prepared Salads and Sandwiches and Dairy products and also in 90.43% of Biscuits, 89.92% of Chocolate and sweets and 67.37% of Crisps and snacks products (Figure 7).

## 4 Discussion

Universities and faculties have long been recognized as institutions that can contribute to promoting health initiatives (34). Recently their role in sustainable development has been increasingly emphasized (35–37) with a particular focus on advocating for sustainable healthy diets (38). Higher education institutions can support healthy and environmentally sustainable eating patterns among students and their employees (39, 40). Within the complex food environment of higher education settings (35), vending machines represent just one option of the many food choices available (36, 37). The latest data on the use of vending machines by students in Slovenia are from 2016, when a Slovene consumer organization conducted a study among more than 1,000 students representing all main Universities. A total of 75% of students bought food on vending machines at least a few times per month, with 10% buying food daily, whereas drinks and sweet snacks are the most frequently purchased products. Only 30% of participating students were satisfied with the offer; others wanted less sweets and more fresh fruits, salads or smoothies (41). Nevertheless, there is a notable absence of strategies addressing the availability, labeling, accessibility, and affordability of healthy and environmentally sustainable foods provided in vending machines (42). By integrating initiatives prioritizing health and environmental considerations, universities and faculties may play a pivotal role in fostering sustainable development within their communities.

Our results support existing research, highlighting a notable scarcity of vending machine products meeting sustainability criteria. Similarly, Bertossi et al. (21) reporting a significant lack of sustainable food products sold at vending machines at the university. While certain categories such as “dairy products,” “fruits,” and “nuts and seeds” demonstrate favorable nutritional profiles, their representation remains minimal, lower than 2.50%. Conversely, products from categories such as biscuits, crisps, snacks, and pre-prepared foods (sandwiches) typically exceed recommended thresholds for fat, salt, and sugar content, posing concerns for cardiovascular health and metabolic diseases (38–40). The results are in line with other studies, reporting the higher representation of nutrient-poor and energy-rich products in vending machines, while more nutritionally favorable products are less frequently available (7, 22, 31, 43, 44).

Production of palm oil is associated with deforestation and negative impacts on the environment (45), it also contains a high amount of saturated fat (46–48). Therefore, the absence of palm oil was identified as one of the criteria in sustainability assessment, yet more than one-quarter of products contained palm oil, and only two were labeled as palm oil-free. Although the presence of palm oil in prepackaged food has been at the center of academic debate for some time (49), it is still present in the majority (more than 50%) of products from the food categories of Chocolates and sweets, Cereal bars and Biscuits offered in the surveyed vending machines.

Many products across categories like biscuits, cereal bars, chocolates, and sweets contain elevated sugar levels, linked to dental caries and metabolic disorders like obesity and type 2 diabetes (50–53). Reducing salt intake is recommended by several dietary guidelines, health organizations, and government policies. Exceeding established salt thresholds, observed across various food categories, underscores the importance of salt reduction initiatives to combat hypertension and related conditions, such as heart failure (49–51). Considering dietary fiber content, particularly some products within the categories of “cereal bars,” “chocolates and sweets,” and “crisps and snacks” could be characterized as sustainable, meaning they contain more than 3.0 g of dietary fiber per 100 g of product and can be labeled in EU as a source of dietary fiber. This aligns with the protective role of dietary fiber intake against colon cancer risk, as suggested by some observational studies (54, 55).

Addressing socioeconomic indicators for sustainable healthy diets, our study noted a limited proportion of products suitable for individuals with special dietary requirements such as gluten sensitivity and lactose intolerance. Gluten and lactose were present in most products from food categories where these two ingredients are expected, although worldwide demand for gluten-free and or lactose-free foods is rising (56, 57). Recognizing consumer preferences and accommodating special dietary needs are important acceptance factors in promoting socioeconomic indicators for sustainable dietary practices (2). This highlights the need for greater diversity and inclusivity in the selection of vending machine products to accommodate various dietary preferences and restrictions.

Sustainable healthy diets need a holistic approach encompassing environmental and socio-cultural dimensions, alongside health and socioeconomic considerations. One of the important impacts of food production on the environment is the use of plastics and derivatives in food packaging. In our assessment only a modest proportion (26.38%) of vending machine products were packaged sustainably, identified as recycled plastic, FSC cardboard, or return packaging. The predominant reliance on conventional food packaging materials underscores significant concerns regarding water use, environmental pollution, and waste production (58, 59). Furthermore, the presence of specific certificates indicative of sustainable sourcing practices, such as Fair-trade cacao, Rainforest Alliance, Cocoa Life, or organic certificates was notably low. These certifications play a crucial role in ensuring ethical and environmentally responsible production practices throughout the supply chain, yet their limited prevalence within vending machine offerings suggests a potential gap in promoting sustainability.

Despite the importance of promoting sustainable and healthy diets, evidenced even within forums like the European Vending Machine Association (21), there remains a notable absence of sustainable food products available through vending machines (19). This deficiency suggests a significant gap in integrating sustainable practices within the vending machine industry. It appears that the agricultural sector is not sufficiently integrated into the vending machine supply chain (20). This lack of involvement from the agricultural stakeholders poses a challenge to sourcing sustainable and locally produced food items for vending machines. In the current survey, only 6 out of 1,213 products were labeled as “Handmade in Slovenia.”

There are a few limitations to this study that could be considered when conducting further research in this field. This are the sample of vending machines, as they were evaluated only at selected locations, however, it should be noted that the offer of vending machines at other faculties in Slovenia does not differ much, as there are only two large vending machine providers on the market. Another limitation of the research is the selection of parameters for assessing sustainable diets, as only some parameters were considered: nutritional value of the products, packaging format, presence of sustainability certificates, presence/absence of palm oil and availability of products for people with special dietary needs, namely gluten sensitivity and lactose intolerance. Although four sustainability criteria were separately considered, the overall assessment of product sustainability was not evaluated. For further research, also the NOVA score could be used to assess the degree of processing, or even LCA score of the products could be determined, but additional data is needed, that was not in the scope of this study.

## 5 Conclusion

This study has highlighted the significant gap between the availability of sustainable, nutritious food options in vending machines at Slovenian universities and the ideal standards for healthy, environmentally responsible diets. Despite the convenience and widespread use of vending machines, our findings suggest that most available food products do not meet the recommended sustainability criteria. This misalignment poses a challenge to promoting healthier eating habits among students and staff, who often rely on these machines for quick meal options.

Our analysis revealed that only a small fraction of the food products met criteria for fat, sugar, salt content, and dietary fiber that align with sustainability. A generally observed limited selection of vending machine offerings labeled as suitable for individuals with special dietary needs (gluten-free, lactose-free) is prompting action toward adjusting offerings for greater dietary diversity and inclusivity. Furthermore, the environmental impact of the packaging materials and the sparse presence of sustainability certifications indicate a broader issue with the current vending machine offerings in terms of environmental responsibility.

Given these findings, there is a clear and urgent need for intervention strategies that could include the implementation of stricter nutritional guidelines, the promotion of healthier and more sustainable food options, and improvements in labeling to make healthier choices more visible and appealing. Universities have a unique position and capability to influence the eating behaviors of many young adults and can serve as a catalyst for change toward more sustainable food consumption patterns. In conclusion, this study underscores the necessity for a coordinated effort among policymakers, educational institutions, and food vendors to enhance the health and sustainability of food offerings in vending machines. Such efforts could significantly contribute to broader public health and environmental sustainability goals, fostering a healthier future for both individuals and the planet.

The authors are currently preparing Recommendations for a healthy choice in vending machines, with included sustainability criteria, which will provide clear guidelines regarding the appropriate offer of food in vending machines at faculties and in health and social care institutions, and will follow the principles of a healthy and sustainable diet.

## Data Availability

The raw data supporting the conclusions of this article will be made available by the authors, without undue reservation.
